# Comparison of three emergency department triage systems among Thai medical students: A randomized three-period crossover simulation study

**DOI:** 10.1371/journal.pone.0350690

**Published:** 2026-10-26

**Authors:** Jarupa Yaowalaorng, Thanin Lokeskrawee, Natthaphon Pruksathorn, Suppachai Lawanaskol, Jayanton Patumanond, Chanitda Wicha, Wanwisa Bumrungpagdee, Suwapim Chanlaor, Chawalit Lakdee

**Affiliations:** 1 Department of Emergency Medicine, Lampang Hospital, Lampang, Thailand; 2 Chaiprakarn Hospital, Chiang Mai, Thailand; 3 Clinical Epidemiology and Clinical Statistics Unit, Faculty of Medicine, Naresuan University, Phitsanulok, Thailand; 4 Department of Radiology, Buddhachinaraj Phitsanulok Hospital, Phitsanulok, Thailand; Universitair Kinderziekenhuis Koningin Fabiola: Hopital Universitaire des Enfants Reine Fabiola, BELGIUM

## Abstract

**Introduction:**

In Thailand, the Emergency Severity Index (ESI) and Ministry of Public Health Emergency Department triage system (MOPH) are commonly used, whereas the Thai Rapid Initial Assessment and Grading for Emergency (TRIAGE) system has been newly proposed. Comparative evidence is limited. We compared correct triage as the primary outcome, with under-triage, over-triage, and completion time as secondary outcomes.

**Methods:**

We conducted a randomized three-period crossover simulation study at the Lampang Medical Education Center from October 2025 to January 2026. Fifth- and sixth-year medical students without recent standardized triage reinforcement classified 50 standardized patient cards per system in one of six sequences, with a one-week washout between periods. Multilevel mixed-effects models were used to estimate between-system differences, with adjustment for period, sequence, and academic year.

**Results:**

Sixty students completed all periods. No significant overall differences were detected in correct triage across TRIAGE, ESI, and MOPH (63.2%, 64.5%, and 61.8%, respectively), or in under-triage (19.6%, 20.0%, and 18.8%). Among sixth-year students, correct triage differed significantly across systems (global P = 0.004), with higher adjusted probabilities for ESI and TRIAGE than MOPH. Over-triage differed across systems and was highest with MOPH (7.0%), followed by TRIAGE (5.7%) and ESI (4.9%). Completion time was longest with TRIAGE (32.8 minutes), compared with ESI (24.3 minutes) and MOPH (23.2 minutes).

**Conclusion:**

No significant overall differences in correct or under-triage were detected, but the study was not powered to establish equivalence. MOPH showed higher over-triage, Completion time was longest with TRIAGE. Generalizability to experienced clinicians requires further study.

**Trial registration:**

Thai Clinical Trials Registry (TCTR) TCTR20260316003

## Introduction

A high-quality emergency department (ED) triage system should be rapid, simple to apply, accurate, and consistent across raters; inter-rater agreement is commonly used as one indicator of reliability [1]. In health professions education, triage represents a complex decision-making competency that requires integration of clinical reasoning, rapid prioritization, and standardized interpretation of patient information. Therefore, evaluating triage performance within a structured educational framework is essential for improving both individual competency and system-level training outcomes.

At Lampang Hospital, the Emergency Severity Index (ESI) was translated into Thai, adapted for local ED triage, and implemented in 2009. In 2012, our team also collaborated with the National Institute for Emergency Medicine (NIEM) to develop a nationwide Thai triage framework. Both the local Lampang system and the national framework were derived from ESI version 4 [1]. For the present study, assessments followed version 5 of the ESI triage algorithm [2], which retains the same core framework while incorporating updates, including more detailed “high-risk” vital sign thresholds stratified by pediatric age groups.

In 2018, the Ministry of Public Health Emergency Department triage (MOPH ED triage; hereafter, MOPH) was introduced as an ESI-based adaptation. This version incorporates blood pressure (BP) thresholds within the algorithm and adds condition-specific descriptors to support clearer differentiation between triage levels [3]. To date, this approach has been rarely described in the international literature, and its comparative performance relative to other systems remains uncertain.

More recently, NIEM convened a national multidisciplinary expert panel comprising emergency physicians (EPs), experienced nurses, and paramedics to develop a draft of the Thai Rapid Initial Assessment and Grading for Emergency (TRIAGE), which has not yet been formally launched. TRIAGE is a criteria-based, rule-driven acuity triage algorithm that integrates physiologic thresholds, symptom severity (pain scale), and early warning tools, including the National Early Warning Score (NEWS) and the Pediatric Early Warning Score (PEWS) (S1 Fig). From an educational perspective, such rule-based systems may influence cognitive load, decision consistency, and learning performance during training.

The co-existence of multiple national and locally adapted triage algorithms in Thailand highlights the need for standardized comparisons within an educational evaluation framework. Direct comparisons of ESI, MOPH, and TRIAGE in routine clinical practice are prone to familiarity and preference effects, which vary across institutions depending on prior exposure. Simulation-based assessment using standardized patient scenarios provides a controlled environment to evaluate performance while minimizing these confounders.

We therefore conducted a randomized crossover study in fifth- and sixth-year medical students without recent standardized triage training immediately before the study. This design allows within-individual comparisons across triage systems and enables robust educational evaluation of performance. This study aimed to compare correct triage across the three systems as the primary outcome and to compare under-triage, over-triage, and completion time as secondary outcomes, with completion time reflecting assessment efficiency.

## Methods

### Ethical statement

This study was approved by the Institutional Review Board of Lampang Hospital, Thailand (original approval, CERT No. 151/68; subsequent administrative amendments, CERT Nos. 151.1/68 and 151.2/68). These administrative amendments concerned only the study title. They did not alter the approved study protocol or the conduct of the study, including the randomized three-period crossover design, participant eligibility and enrollment, randomization and allocation procedures, washout interval, standardized assessment procedures, blinding to the reference classifications, or participant protections. Written informed consent was obtained from all participants before enrollment. As medical students may be considered a potentially vulnerable population within an educational setting, participation was entirely voluntary, and eligible students were free to decline without academic obligation or coercion. Participation was not mandated by teaching staff and had no influence on academic evaluation, grading, attitude assessment, or clinical rotation outcomes. The study was registered with the Thai Clinical Trials Registry (TCTR20260316003) on 16 March 2026, after completion of data collection. Prospective registration was not undertaken because, at the time of study initiation, the registry did not permit amendments to an existing record, and subsequent protocol changes would have required a new registration number. This was a simulation-based educational study using standardized fictional patient cards; no real patients were involved, and no treatment or clinical intervention was evaluated. The study was conducted in accordance with the principles of the Declaration of Helsinki and reported in accordance with the CONSORT extension for crossover trials [4].

### Study design

A randomized three-period crossover design was used to compare three ED triage systems through a standardized simulation-based assessment. This design allowed direct within-participant comparisons across triage systems, thereby reducing between-participant variability. The study comprised three sequential periods, each corresponding to one triage system. The order of triage systems was determined by random assignment to one of six possible sequences to distribute potential order effects across participants. Blinding to the reference answers was maintained throughout the crossover, and feedback was withheld until completion of the study. Assessment difficulty was standardized across the three patient-card sets to support fair comparison of performance across periods. Potential carryover effects were addressed through design-based measures, including a one-week washout interval between consecutive periods [5].

### Setting and participants

This study was conducted at the Lampang Medical Education Center from 1 October 2025 to 31 January 2026. Eligible participants were fifth- and sixth-year medical students without recent standardized triage training immediately before the study, hereafter referred to as students without recent triage reinforcement. Exclusion criteria were declining to participate, being unavailable during the study period, or being unable to commit to participation in all three crossover periods.

### Study procedures

The study procedures consisted of applying three ED triage systems—TRIAGE, ESI, and MOPH—in a randomized three-period crossover sequence, with one system used per period. During each period, the assigned triage system was displayed on a computer screen, including the system-specific algorithm and on-screen prompts. Participants could not access or view the algorithms of the other triage systems during each assessment period, thereby preventing concurrent exposure to the nonassigned systems. To maintain the no-recent-triage-reinforcement condition throughout the study, no teaching sessions or supplementary instruction were provided, and participants received no additional guidance or clarification regarding triage decision-making at any stage. No feedback was provided; correct triage classifications were withheld until all three periods were completed.

Standardized patient cards were used as the assessment material [6]. Each card presented structured information in a consistent format, including a brief history, key symptoms, primary survey findings, vital sign parameters, and a pain scale. An animated patient figure illustrated sex, age, appearance, and mode of mobility (walk-in, wheelchair, or stretcher), aligned with the clinical details displayed on the right (S2 Fig).

The standardized patient cards were derived from a pool of 300 triage case cards previously used in a routine triage-training program comprising 10 assessments of 30 cards each. Three experienced triage nurses with the highest concordance with the reference answers established by two senior EPs were retrospectively identified. Their item-level response patterns across the pooled cards were subsequently used to classify card difficulty. A card was categorized as easy when all three nurses assigned the correct triage level, intermediate when two assigned the correct level and one assigned a different level, and hard when the three nurses assigned three different triage levels. For example, when the correct triage level was 2, response patterns of 2/2/2, 1/2/2, and 1/2/3 were categorized as easy, intermediate, and hard, respectively.

The 300 difficulty-labeled cards were then randomly sampled within each difficulty category to construct three distinct 50-card test sets, comprising 150 unique study cards in total. Each set was designed to contain an approximately equivalent distribution of 85% easy, 10% intermediate, and 5% hard cards. The difficulty distributions were comparable across the three test sets (S1 Table; P = 0.998). Within each period, participants were randomly assigned one of the three test sets, with a program-based restriction preventing reuse of the same set by the same participant across periods. The order of cards within each assigned set was randomly shuffled. The three nurses involved in difficulty classification were distinct from the experts responsible for establishing the system-specific reference answers, as described in the subsequent section.

During each study period, participant responses were recorded and processed using the Thailand Triage Training Kit (TTTK) software [7] according to the triage system allocated for that period. Raw data were subsequently extracted and cleaned before statistical analysis. Apart from the triage system and test set assigned to each participant within period, all study procedures and assessment conditions were standardized across periods to ensure consistency in performance evaluation.

### Reference standard and outcome assessment

The experts who established the system-specific reference classifications were entirely distinct from the three nurses who assessed card difficulty. For each triage system, an independent expert panel comprising three EPs from different hospitals established the reference classifications for all 150 standardized patient cards. Each panel represented the respective committee or expert group responsible for the development, implementation, or governance of the assigned triage system (TRIAGE, ESI, or MOPH). All adjudicators independently assigned a triage level to each patient card according to the predefined criteria of the respective system. The final reference classification was determined using a two-out-of-three majority rule. No card received three different classifications. When one adjudicator differed from the other two, the differing classification was no more than one acuity level from the majority classification. Therefore, no consensus discussion or card exclusion was required. Interrater agreement was evaluated using Conger’s linear weighted kappa [8,9] and demonstrated excellent agreement across all three expert panels (S2 Table).

All three triage systems use five acuity levels, with level 1 representing life-threatening conditions requiring immediate intervention and level 5 representing the lowest-acuity conditions. Each participant-assigned triage level was compared with the corresponding system-specific reference classification.

The primary outcome was correct triage classification, defined as concordance between the participant-assigned triage level and the corresponding reference classification. Correct triage was expressed as the percentage of patient-card assessments classified correctly.

The secondary safety outcomes were under-triage and over-triage. Under-triage was defined as assignment of a less urgent triage level than the corresponding reference classification, whereas over-triage was defined as assignment of a more urgent triage level than the corresponding reference classification. Both outcomes were expressed as percentages of patient-card assessments.

The secondary efficiency outcome was completion time, defined as the total time, in minutes, required for each participant to complete all 50 patient cards during each study period. Unlike the card-level classification outcomes, completion time was assessed at the participant-period level.

### Harms

As this was a simulation-based study without clinical intervention, no adverse event monitoring was conducted.

### Study size estimation

Given the absence of published comparative data for ESI, MOPH, and TRIAGE among medical students without recent standardized triage reinforcement, a separate pilot study in the target population was not conducted to avoid educational contamination and preserve the limited pool of eligible students during clinical rotations.

For initial study planning, we used the best available pilot data from a simulation-based assessment involving 40 triage nurses who evaluated patient cards developed using the same standardized framework. The observed overall accuracy was 65.0% for TRIAGE, 70.0% for ESI, and 68.5% for MOPH. Although the pilot population differed from the target population, these data were considered the most relevant available estimates because comparative evidence for MOPH was limited and TRIAGE had only recently been proposed.

As the protocol-defined primary outcome was binary classification accuracy assessed repeatedly within participants, McNemar’s test for paired proportions was used as an approximate initial planning method. The smallest anticipated difference was between ESI and MOPH, for which the calculation indicated that 7,433 paired scenario evaluations would be required. Assuming that each participant contributed 50 classification assessments under each system, this corresponded to approximately 149 participants for a conventional pairwise comparison. This calculation was treated as an approximate planning estimate because it did not fully account for the crossover design, clustering of assessments within participants and patient cards, or the use of different patient-card sets across study periods rather than assessment of the same cards under each system.

### Interim analysis

No interim analyses or stopping guidelines were planned for this simulation-based crossover study. Interim analyses were not conducted, as doing so could introduce operational bias and potentially influence study conduct, data interpretation, or participant performance in subsequent periods. The study therefore proceeded to the prespecified target sample size to preserve statistical power and precision.

### Randomization, allocation concealment, and implementation

The random allocation sequence was generated in advance using computer-based simple randomization. Participants were assigned to one of the six possible sequences of the three triage systems in the three-period crossover design. No stratification was applied because each participant was scheduled to assess all three triage systems, thereby supporting within-participant comparisons across systems.

The allocation sequence was generated by research center staff who were not involved in participant enrollment, assessment, or data analysis. Participants were enrolled by officers of the Lampang Medical Education Center and assigned to one of the six predefined sequences according to the randomization list. This list was inaccessible to the officers responsible for participant enrollment, thereby maintaining allocation concealment until sequence assignment.

Participants were not informed in advance of the triage systems assigned to subsequent study periods. At the beginning of each period, the study coordinator disclosed and verified the assigned system immediately before the assessment to minimize misassignment.

### Blinding and standardization of assessment conditions

The study was conducted in a simulation room accommodating up to 12 participants per session; therefore, five rounds were required in each study period. Participants were seated with adequate spacing to prevent visual sharing of responses. Although assessments were conducted simultaneously, each participant used the assigned triage system according to the randomized sequence and was not informed of the system used by adjacent participants.

Three distinct randomization procedures were used. First, participants were randomly assigned to one of the six possible sequences of TRIAGE, ESI, and MOPH, as described above. Second, independently of the triage-system sequence, one of the three 50-card sets was randomly assigned to each participant in each period. The computerized system prevented any participant from receiving the same card set more than once. Consequently, each participant completed all three card sets without repetition. Third, the order of the 50 cards within each assigned set was independently shuffled for each participant. Card numbers were hidden to prevent participants from determining whether adjacent participants were assessing the same case, remembering specific card numbers from earlier sessions, or following the progress of others. These procedures reduced opportunities for copying, influence between participants, case memorization, and repeated exposure to the same card set. The three card sets had comparable overall difficulty, supporting standardized assessment conditions.

### Statistical analysis

Baseline characteristics were summarized descriptively. Categorical variables were presented as counts and percentages. Continuous variables were summarized as mean ± standard deviation (SD) or median (interquartile range [IQR]), depending on their distribution.

Outcomes were analyzed using multilevel regression models with random intercepts, appropriate to the outcome scale. The main comparison was triage system, which was specified as the primary fixed-effect term. All adjusted models also included study period, allocation sequence, and academic year. For card-level classification outcomes, multilevel logistic regression models with random intercepts for participant and patient card were fitted separately for correct triage classification, under-triage, and over-triage to account for repeated assessments within participants and differences in card difficulty. For completion time, a multilevel linear regression model was fitted at the participant-period level, with a random intercept for participant to account for repeated completion-time measurements within participants.

Adjusted marginal estimates were calculated from each model and presented with 95% confidence intervals (CIs); estimates for classification outcomes and completion time were reported as percentages and minutes, respectively. Overall differences among the three triage systems were assessed using Wald tests [10]. Prespecified pairwise comparisons included TRIAGE versus MOPH, ESI versus MOPH, and TRIAGE versus ESI. To account for multiple pairwise testing across the three planned comparisons, Bonferroni-adjusted P values were reported [11,12]. A Bonferroni-adjusted P value < 0.05 was considered statistically significant.

Model convergence and variance component estimates were examined to confirm model stability. All statistical analyses were performed using Stata v18 (StataCorp, College Station, TX, USA; licensed).

### Additional analyses

A prespecified subgroup comparison stratified by academic year (fifth vs. sixth year) was conducted for correct, under-, and over-triage classifications to assess whether the relative performance of the three ED triage systems was consistent between the two student groups. Restricting this comparison to the classification outcomes improved interpretability and limited multiplicity. Completion time was not included in the prespecified subgroup assessment and was evaluated only through overall marginal comparisons.

Additional post hoc exploratory evaluations examined temporal robustness and crossover-related effects, including period effects, system-by-period interactions, and carryover from the system used in the preceding period. Robustness was further assessed using data restricted to period 1 and to periods 1 and 2. These assessments covered all three classification outcomes and completion time.

## Results

Recruitment and outcome assessment were conducted from October 2025 to January 2026. Ninety-seven medical students were assessed for eligibility (fifth-year, n = 42; sixth-year, n = 55). Thirty-seven students were excluded before randomization: five declined to participate, 27 were unavailable during the study period, and five were unable to commit to participation across all three study periods. A total of 60 participants provided informed consent and were randomly assigned to one of the six crossover sequences: sequence A, n = 10; sequence B, n = 9; sequence C, n = 9; sequence D, n = 9; sequence E, n = 12; and sequence F, n = 11. All 60 participants completed the three study periods, with no losses to follow-up or post-randomization exclusions.

Each participant completed three assessment periods, one for each triage system, with a one-week washout interval between periods. In each period, participants evaluated 50 standardized patient cards, resulting in 180 participant-period assessments (60 × 3) and 9,000 total patient-card assessments (60 × 3 × 50) (Fig 1).

**Fig 1:**
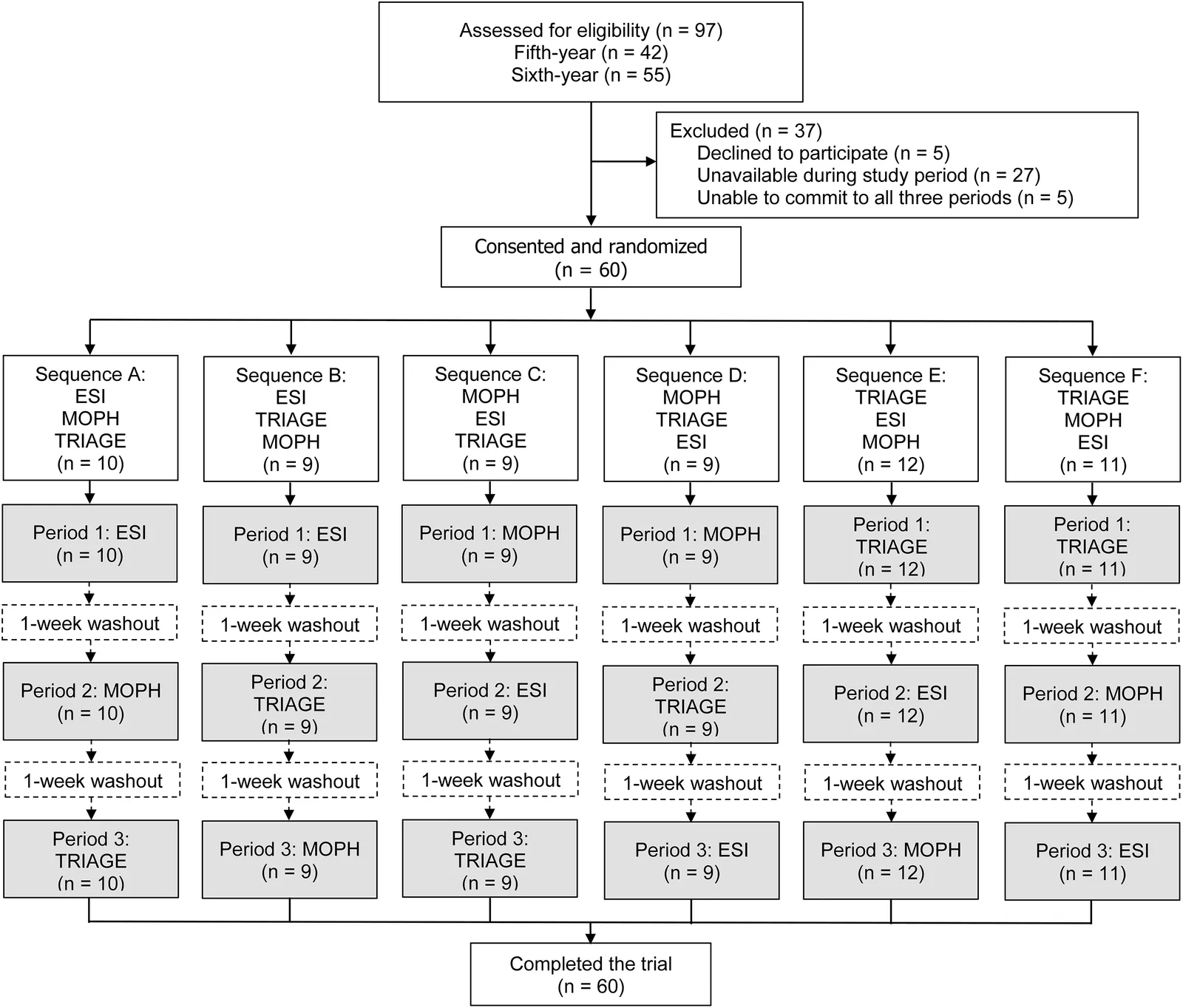
Study flow diagram. Participants were fifth- and sixth-year medical students at the Lampang Medical Education Center who were enrolled between October 2025 and January 2026.

Of the 60 participants, 51.7% were female, and the mean age was 23 years. Fifth- and sixth-year students were equally represented (30 each) (Table 1).

**Table 1:** Baseline characteristics of participants in the randomized three-period crossover study.

Characteristics	N = 60, n (%)
Female	31 (51.7)
Age (years), mean ± SD	23.0 ± 0.7
Academic year	
Fifth year	30 (50.0)
Sixth year	30 (50.0)

Only selected baseline characteristics were collected for this simulation-based crossover assessment.

Across study periods, crude correctness generally declined for all three triage systems. TRIAGE showed the largest decrease, from 68.5% in period 1 to 52.6% in period 3. ESI increased modestly from 62.2% in period 1 to 65.6% in period 2 before declining to 59.7% in period 3, whereas MOPH decreased from 63.3% in period 1 to 58.2% in period 3 (Table 2).

**Table 2:** Crude correctness by study period and triage system.

Triage system	Correctness	Period 1, n/N (%)	Period 2, n/N (%)	Period 3, n/N (%)	Overall, n/N (%)
TRIAGE	Correct	788/1,150 (68.5)	560/900 (62.2)	500/950 (52.6)	1,848/3,000 (61.6)
	Incorrect	362/1,150 (31.5)	340/900 (37.8)	450/950 (47.4)	1,152/3,000 (38.4)
ESI	Correct	591/950 (62.2)	689/1,050 (65.6)	597/1,000 (59.7)	1,877/3,000 (62.6)
	Incorrect	359/950 (37.8)	361/1,050 (34.4)	403/1,000 (40.3)	1,123/3,000 (37.4)
MOPH	Correct	570/900 (63.3)	623/1,050 (59.3)	611/1,050 (58.2)	1,804/3,000 (60.1)
	Incorrect	330/900 (36.7)	427/1,050 (40.7)	439/1,050 (41.8)	1,196/3,000 (39.9)

Data are presented as n/N, with percentages in parentheses, where n represents the number of correct or incorrect patient-card assessments and N represents the total number of patient-card assessments for each triage system within each period or overall. TRIAGE: Thai Rapid Initial Assessment and Grading for Emergency; ESI: Emergency Severity Index; MOPH: Ministry of Public Health Emergency Department Triage.

After adjustment for period, sequence, and academic year, correct-triage estimates ranged from 61.8% to 64.5% across the three systems, with no significant overall difference (global P = 0.124) or Bonferroni-adjusted pairwise differences (all adjusted P ≥ 0.124). Under-triage estimates ranged from 18.8% to 20.0%, with no significant overall or pairwise differences (global P = 0.449; all adjusted P ≥ 0.652). In contrast, adjusted over-triage differed significantly among the systems (global P < 0.001). MOPH had the highest adjusted probability at 7.0%, compared with 5.7% for TRIAGE and 4.9% for ESI. Over-triage was significantly lower with TRIAGE than with MOPH (adjusted P = 0.043) and with ESI than with MOPH (adjusted P = 0.001), whereas TRIAGE and ESI did not differ significantly (adjusted P = 0.239) (Table 3).

**Table 3:** Adjusted probabilities of correct triage, under-triage, and over-triage by triage system.

Panel A. Correct triage				
Triage system	Adjusted probability, % (95% CI)	Global P value	vs. MOPH P value^†^	vs. ESI P value^†^
TRIAGE	63.2 (59.1–67.3)	0.124	0.889	0.968
ESI	64.5 (60.5–68.5)		0.124	Ref
MOPH	61.8 (57.7–66.0)		Ref	–
Panel B. Under-triage				
Triage system	Adjusted probability, % (95% CI)	Global P value	vs. MOPH P value^†^	vs. ESI P value^†^
TRIAGE	19.6 (15.9–23.4)	0.449	1.000	1.000
ESI	20.0 (16.2–23.9)		0.652	Ref
MOPH	18.8 (15.1–22.4)		Ref	–
Panel C. Over-triage				
Triage system	Adjusted probability, % (95% CI)	Global P value	vs. MOPH P value^†^	vs. ESI P value^†^
TRIAGE	5.7 (3.6–7.8)	< 0.001	0.043	0.239
ESI	4.9 (3.0–6.7)		0.001	Ref
MOPH	7.0 (4.5–9.6)		Ref	–

Percentages are estimated marginal probabilities from separate multilevel mixed-effects logistic regression models for correct triage, under-triage, and over-triage, adjusted for period, sequence, and academic year, with crossed random intercepts for participant and patient card. Because the three outcomes were modeled separately, the estimated probabilities are not constrained to sum to 100%. ^†^Bonferroni-adjusted P value. CI: confidence interval; TRIAGE: Thai Rapid Initial Assessment and Grading for Emergency; ESI: Emergency Severity Index; MOPH: Ministry of Public Health Emergency Department Triage.

The full 5 × 5 distributions of participant-assigned versus reference-standard triage levels comprised 3,000 card-level assessments per system and 9,000 assessments overall (S3a–S3c Table).

The adjusted pairwise differences in correct triage were small, ranging from −1.15 to 2.37 percentage points. Similarly, differences in under-triage ranged from −0.39 to 1.23 percentage points; all 95% CIs included zero (Table 4).

**Table 4:** Adjusted pairwise differences in correct triage and under-triage between triage systems.

Outcome	Comparison	Adjusted difference, percentage points (95% CI)	Adjusted P value^†^
Correct triage	TRIAGE vs. MOPH	+1.22 (−1.58 to 4.02)	0.889
	ESI vs. MOPH	+2.37 (−0.41 to 5.15)	0.124
	TRIAGE vs. ESI	−1.15 (−3.94 to 1.64)	0.968
Under-triage	TRIAGE vs. MOPH	+0.84 (−1.50 to 3.18)	1.000
	ESI vs. MOPH	+1.23 (−1.15 to 3.60)	0.652
	TRIAGE vs. ESI	−0.39 (−2.76 to 1.98)	1.000

Adjusted differences are pairwise differences in estimated marginal probabilities (first-listed system minus second-listed system) from separate multilevel mixed-effects logistic regression models for correct triage and under-triage, adjusted for period, sequence, and academic year, with crossed random intercepts for participant and patient card. Positive values indicate a higher adjusted probability in the first-listed system. ^†^Bonferroni-adjusted P value. CI: confidence interval; TRIAGE: Thai Rapid Initial Assessment and Grading for Emergency; ESI: Emergency Severity Index; MOPH: Ministry of Public Health Emergency Department Triage.

Across the three study periods, the systems showed broadly similar temporal patterns. Correct triage declined by period 3, accompanied by increases in under-triage and over-triage. ESI tended to show the highest correct-triage and lowest over-triage estimates, whereas MOPH generally showed the opposite pattern (Fig 2).

**Fig 2:**
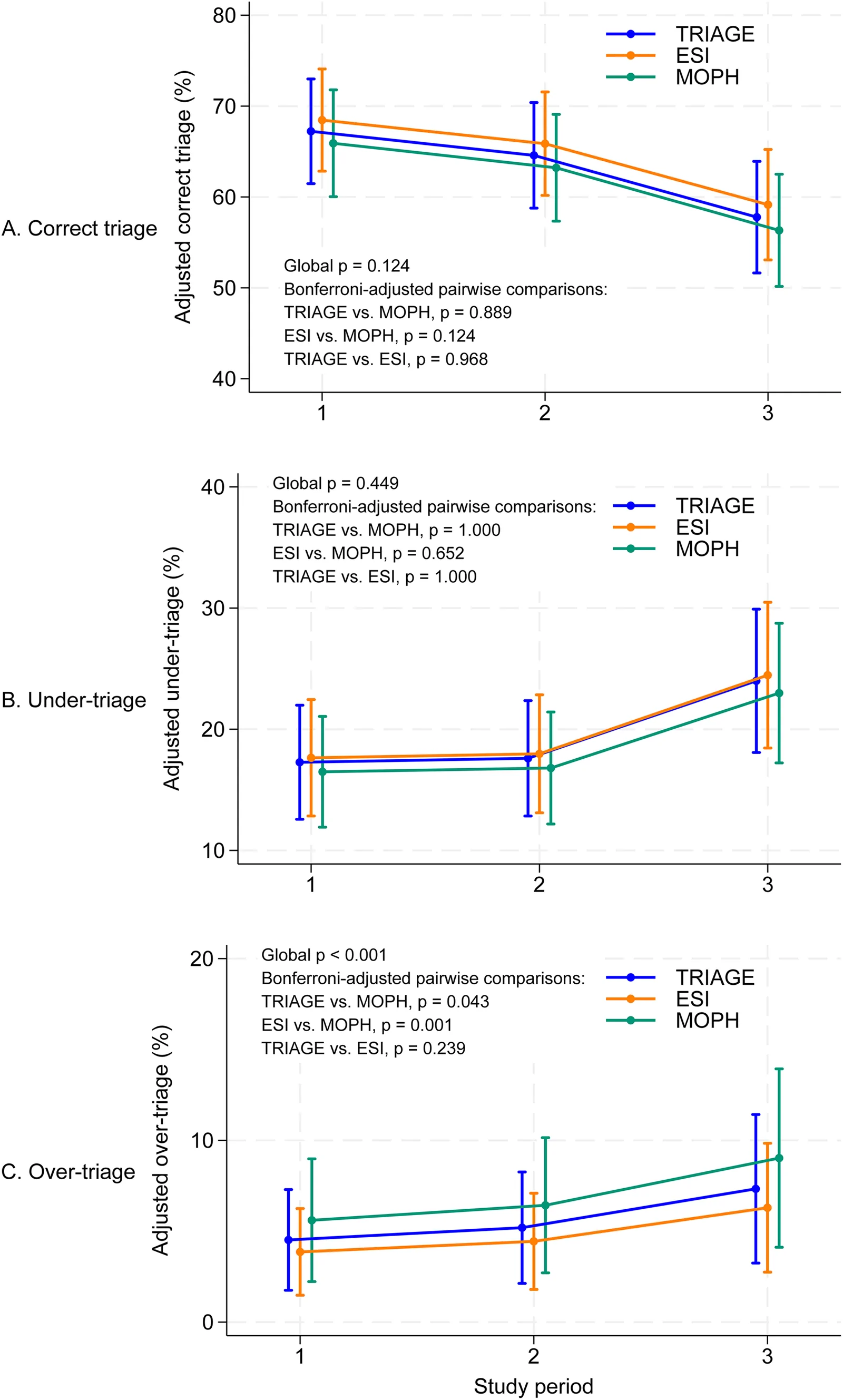
Adjusted marginal percentages of correct triage (A), under-triage (B), and over-triage (C) for TRIAGE, ESI, and MOPH across study periods. Points indicate adjusted estimates, and error bars indicate 95% confidence intervals. TRIAGE: Thai Rapid Initial Assessment and Grading for Emergency; ESI: Emergency Severity Index; MOPH: Ministry of Public Health Emergency Department triage.

Crude completion time decreased across periods for all three systems. TRIAGE consistently required the longest completion time, decreasing from approximately 38 minutes in period 1 to about 30 minutes thereafter, whereas ESI and MOPH were consistently faster and approached 20 minutes by period 3 (Table 5).

**Table 5:** Crude completion time by triage system and study period.

Period	Crude completion time (minutes)						P value
	TRIAGE		ESI		MOPH		
	n	Mean ± SD	n	Mean ± SD	n	Mean ± SD	
1	23	37.9 ± 10.9	19	29.1 ± 6.9	18	27.8 ± 5.1	< 0.001
2	18	30.9 ± 8.6	21	23.3 ± 4.6	21	22.1 ± 3.8	< 0.001
3	19	29.5 ± 5.1	20	20.5 ± 3.7	21	19.6 ± 3.6	< 0.001

TRIAGE: Thai Rapid Initial Assessment and Grading for Emergency; ESI: Emergency Severity Index; MOPH: Ministry of Public Health Emergency Department triage system; n: number of participant-period observations contributing completion time; SD: standard deviation.

After adjustment for period, sequence, and academic year, mean completion time differed significantly among the three systems (global P < 0.001). TRIAGE consistently required significantly more time than both MOPH and ESI (both Bonferroni-adjusted P < 0.001), whereas completion times did not differ significantly between ESI and MOPH (adjusted P = 0.907) (Table 6).

**Table 6:** Adjusted mean completion time by triage system.

Triage system	Adjusted mean completion time (minutes) (95% CI)	Global P value	vs. MOPH P value^†^	vs. ESI P value^†^
TRIAGE	32.8 (31.3–34.3)	< 0.001	< 0.001	< 0.001
ESI	24.3 (22.8–25.8)		0.907	Ref
MOPH	23.2 (21.7–24.7)		Ref	–

TRIAGE: Thai Rapid Initial Assessment and Grading for Emergency; ESI: Emergency Severity Index; MOPH: Ministry of Public Health Emergency Department triage system; CI: confidence interval. Adjusted mean completion times were estimated using multilevel linear models with period effect, sequence effect, and academic year as covariates. ^†^Bonferroni-adjusted P value.

Across study periods, completion time decreased for all three systems, with the greatest reduction occurring between periods 1 and 2, followed by a smaller decline through period 3. The relative pattern remained consistent: TRIAGE required the longest time throughout, whereas ESI and MOPH followed closely similar trajectories at lower completion times (Fig 3).

**Fig 3:**
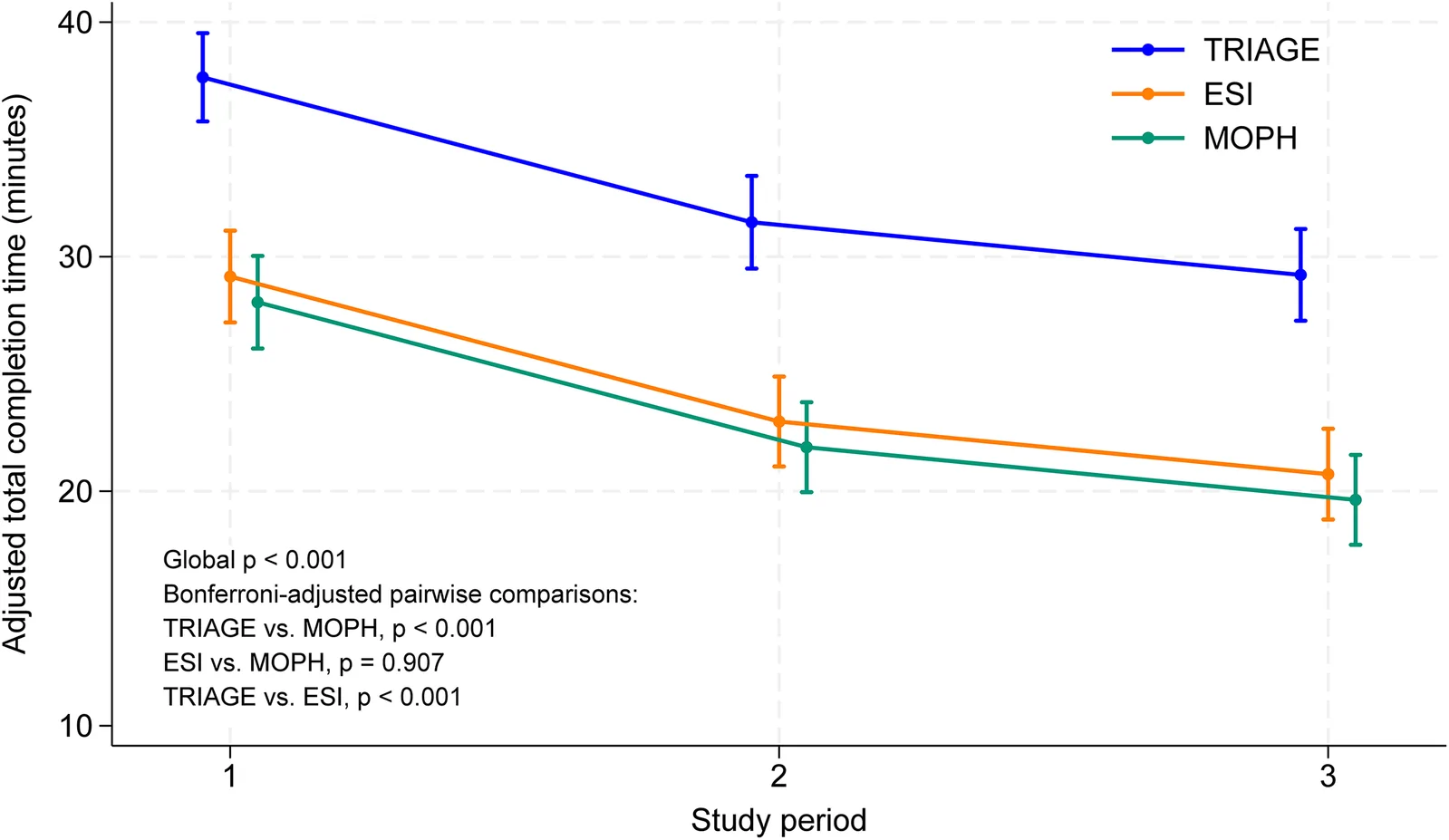
Adjusted marginal mean completion time for TRIAGE, ESI, and MOPH across study periods. Points indicate adjusted estimates, and error bars indicate 95% confidence intervals. TRIAGE: Thai Rapid Initial Assessment and Grading for Emergency; ESI: Emergency Severity Index; MOPH: Ministry of Public Health Emergency Department triage.

## Discussion

### Interpretation

In the overall cohort, no statistically significant differences in correct- or under-triage classifications were detected across TRIAGE, ESI, and MOPH; however, MOPH showed higher over-triage, whereas TRIAGE required longer completion time than ESI and MOPH. Faster completion is less meaningful when assigned triage levels do not align with the corresponding reference classifications. As each system was evaluated against its own reference standard, the observed correct- and under-triage findings indicate students’ fidelity in applying the respective algorithms rather than equivalent clinical validity or superiority of one system over another. These findings are limited to a standardized educational simulation and may not extend to experienced healthcare professionals performing triage in routine clinical practice.

Although Lampang Hospital and Chiang Mai University share examinations, grading, and degree requirements [13], their EDs use different triage systems. The institutions therefore agreed to exclude triage from both multiple-choice and modified essay examinations, ensuring that triage performance did not affect academic grades or promote preparation for a specific system. Fifth-year students consequently received no structured triage training before the study, whereas sixth-year students had received no recent reinforcement. Their last ED rotation preceded data collection by approximately eight months, beyond the six-month interval over which triage performance may decline without continued practice or reinforcement [14]. To prevent educational contamination, triage instruction and case review were deferred until data collection was completed; thereafter, all participants received structured training to ensure that they acquired knowledge applicable to future clinical practice.

Medical students were intentionally selected because this educational setting permitted close characterization of prior exposure and strict control of system-specific training before participation. Achieving a similar level of standardization would have been more difficult among nursing students, whose undergraduate curricula, timing and depth of triage-related instruction, as well as clinical exposure, were less well characterized by the investigators. Triage competency among nurses may also continue to develop during the first 2–3 years after graduation [15]. A further concern is that nursing students could have introduced greater heterogeneity in baseline knowledge and clinical experience, thereby reducing the clarity of between-system comparisons.

The prespecified subgroup analysis detected no significant between-system differences in classification outcomes among fifth-year students. For sixth-year students, correct triage differed significantly across systems, with ESI and TRIAGE showing higher adjusted probabilities than MOPH, whereas under-triage did not differ significantly. Over-triage also differed, with ESI showing the lowest estimate and MOPH the highest (Table 7). The period-specific trajectories were closely aligned across systems among fifth-year students, whereas clearer separation was observed for sixth-year students, with ESI generally showing higher correct-triage and lower over-triage estimates and MOPH showing the opposite pattern (Fig 4). These findings suggest that the between-system differences in correct triage within the sixth-year subgroup were reflected mainly in over-triage.

**Table 7:** Prespecified subgroup analysis of adjusted marginal probabilities of correct triage, under-triage, and over-triage across the three triage systems, stratified by academic year.

Academic year	Outcomes	TRIAGE, % (95% CI)	ESI, % (95% CI)	MOPH, % (95% CI)	P value			
					Global	TRIAGE vs. MOPH^†^	ESI vs. MOPH^†^	TRIAGE vs. ESI^†^
6^th^ year	Correct triage	65.7 (61.0–70.4)	67.0 (62.3–71.6)	61.1 (56.2–66.0)	0.004	0.038	0.005	1.000
	Under-triage	19.0 (14.7–23.4)	19.2 (14.7–23.6)	19.0 (14.6–23.4)	0.992	1.000	1.000	1.000
	Over-triage	5.8 (3.5–8.1)	3.9 (2.3–5.5)	7.5 (4.6–10.4)	< 0.001	0.087	< 0.001	0.013
5^th^ year	Correct triage	60.3 (54.6–66.1)	60.3 (54.6–66.0)	61.3 (55.6–66.9)	0.849	1.000	1.000	1.000
	Under-triage	21.6 (16.7–26.4)	21.6 (16.9–26.4)	20.0 (15.5–24.6)	0.483	0.940	0.848	1.000
	Over-triage	6.8 (4.3–9.2)	7.4 (4.6–10.1)	7.9 (5.0–10.9)	0.332	0.473	1.000	1.000

TRIAGE: Thai Rapid Initial Assessment and Grading for Emergency; ESI: Emergency Severity Index; MOPH: Ministry of Public Health Emergency Department triage system; CI: confidence interval. Percentages are estimated marginal probabilities from multilevel logistic models adjusted for period effect and sequence effect. ^†^Bonferroni-adjusted P value.

**Fig 4:**
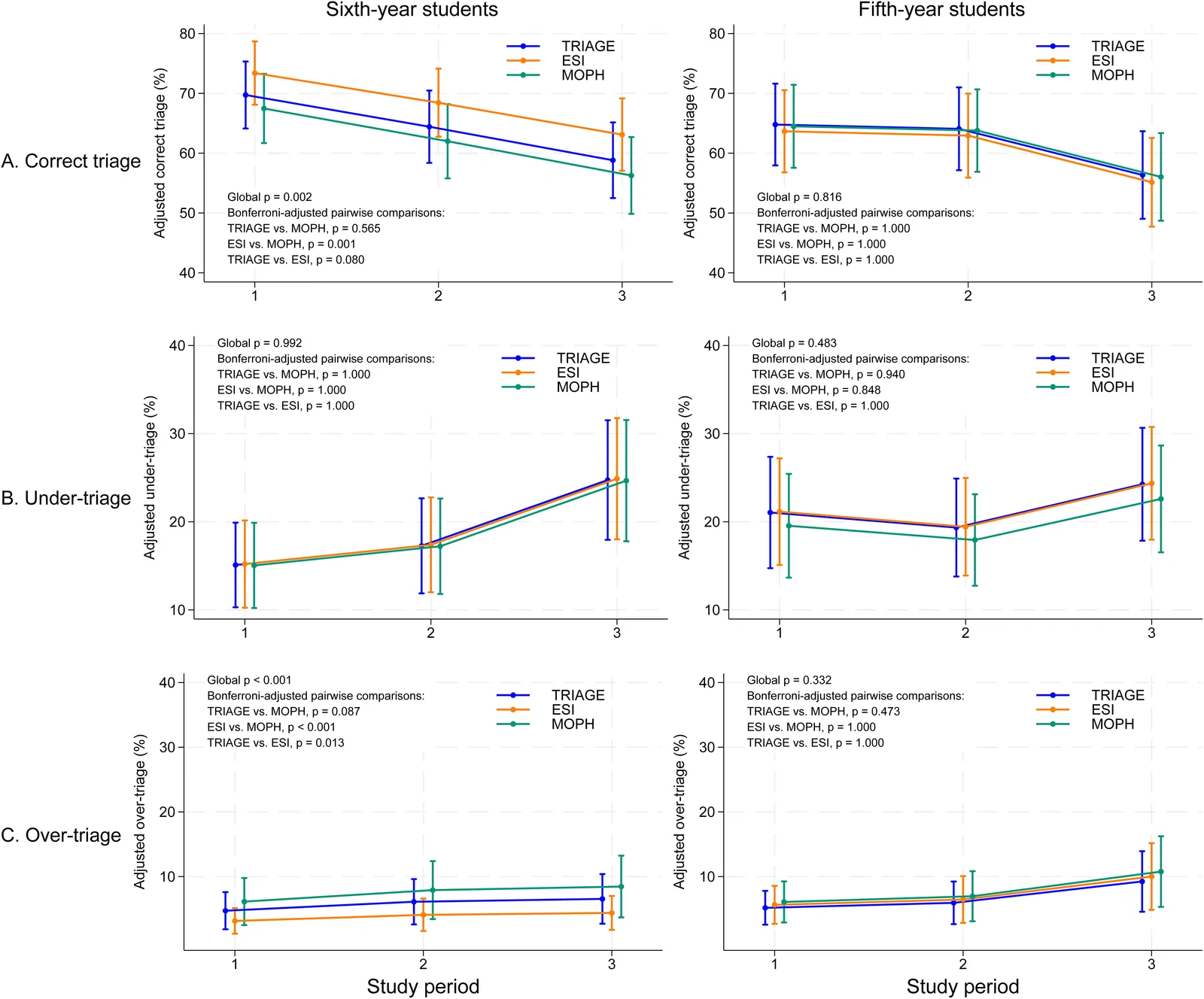
Adjusted marginal percentages of correct triage (A), under-triage (B), and over-triage (C) for TRIAGE, ESI, and MOPH across study periods, stratified by academic year. Points indicate adjusted estimates, and error bars indicate 95% confidence intervals. TRIAGE: Thai Rapid Initial Assessment and Grading for Emergency; ESI: Emergency Severity Index; MOPH: Ministry of Public Health Emergency Department triage.

Although the study design strictly minimized educational contamination, incidental background exposure to MOPH could not be fully controlled because its materials were publicly available and accessible outside the study. Greater familiarity with MOPH criteria may have led participants to assign borderline cases to a more urgent triage level, thereby contributing to higher over-triage. This explanation remains speculative, as prior familiarity and adherence to individual algorithm steps were not directly measured.

A crossover design was considered particularly appropriate for this educational simulation because each participant was evaluated using all three triage systems, allowing direct within-participant comparisons. Unlike a conventional parallel-group randomized trial, in which each participant would be exposed to only one triage system, the crossover approach enabled every participant to experience TRIAGE, ESI, and MOPH while serving as their own control. This design reduces between-participant variability arising from differences in baseline knowledge, clinical reasoning, and decision-making style, thereby improving the precision of comparisons within the available sample. The validity of this approach, however, depends on minimizing residual effects between study periods, making assessment of carryover an important component of the analysis.

Exploratory crossover-related analyses showed no measurable carryover for any classification outcome or completion time, suggesting that the one-week interval was adequate to limit detectable residual effects between study periods. Under-triage was the most temporally stable outcome, with neither a period effect nor a system-by-period interaction, indicating that its overall level and between-system pattern remained broadly unchanged throughout the study. This stability may reflect the novice learner population, in which participants without recent structured triage reinforcement may have preferentially classified uncertain cases into higher-acuity categories rather than risk under-triage. Correct triage showed both an overall period effect and a system-by-period interaction, whereas over-triage varied differently across systems over time, suggesting that the observed between-system differences were partly period-dependent rather than constant throughout the study (S4 Table).

Completion time decreased across study periods for all three systems, indicating increasing familiarity with the standardized patient-card format and the TTTK software used for assessment. The significant system-by-period interaction further showed that the magnitude of this improvement differed among systems, reflecting distinct temporal patterns in completing each algorithm (S4 Table).

Importantly, between-system differences in completion time were already present in the first-period-only analysis and remained after exclusion of period 3, with TRIAGE consistently requiring the longest completion time (S5 Table). These findings indicate that the triage algorithm itself contributed materially to completion time, rather than the observed differences being explained primarily by increasing familiarity across periods. TRIAGE’s longer completion time likely reflects its greater number of decision nodes, more sequential assessment steps, higher cognitive processing demands, and less streamlined workflow.

### Limitations

This study has several limitations. First, it was conducted among medical students using standardized simulated patient cards; therefore, the findings primarily reflect initial algorithm application in a controlled educational simulation and may not generalize to practicing nurses, physicians, or real-world ED triage. Each system was also evaluated against its own system-specific reference classification rather than a common external standard of true clinical urgency. Accordingly, the lack of a significant difference in correct triage should be interpreted in terms of performance in applying the respective algorithms, not as evidence of equivalent clinical validity among the three systems.

Second, background exposure to MOPH could not be completely excluded because its materials were publicly available, particularly among sixth-year students with previous clinical experience. Prior familiarity with TRIAGE was effectively absent because the system had not yet been launched or implemented. ESI had not been formally taught to fifth-year students, whereas sixth-year students may have encountered it during an earlier ED rotation. Despite the approximately eight-month interval since that rotation, residual memory of prior ESI exposure could not be completely excluded. As individual exposure to ESI or MOPH outside the formal curriculum was not directly measured, its possible contribution to the observed classification patterns, particularly the higher over-triage with MOPH, remains uncertain.

Third, reasonable participant compensation was provided to support retention across all three study periods, and no loss to follow-up occurred. Although compensation may have helped maintain engagement, motivation was not directly measured and could not be assumed to remain consistent throughout the repeated assessments. Therefore, reduced motivation or attention over time could not be excluded as a possible contributor to faster completion and lower classification performance.

Fourth, card difficulty was calibrated using the responses of three experienced triage nurses. Cases classified as easy for experienced nurses may not have been equally easy for medical students, given differences in clinical experience and triage expertise. Therefore, the nurse-based difficulty categories may not have fully represented the perceived or actual difficulty of the patient cards in the study population. Although the three card sets had comparable nurse-rated difficulty distributions, residual differences in student-level difficulty across sets could not be completely excluded.

Fifth, sample-size planning relied on pilot data from emergency nurses rather than medical students. A separate pilot study in the target population was intentionally avoided to reduce educational contamination and preserve the limited eligible student pool. As the nurse pilot population differed from the target medical-student population, the expected between-system differences may not have been estimated precisely. Furthermore, only 60 participants were enrolled, fewer than the approximately 149 indicated by the strict initial pairwise calculation. The study size was also not planned specifically for subgroup or sensitivity analyses; therefore, nonsignificant findings and exploratory analyses should be interpreted cautiously.

Sixth, simple randomization resulted in unequal numbers of participants across the six crossover sequences. Although sequence was adjusted for as a fixed effect in the analyses, permuted-block randomization would likely have achieved more balanced allocation and should be considered in future crossover studies.

Seventh, the study classified outcomes as correct, under-, or over-triage without further accounting for the direction or magnitude of misclassification. Errors could occur toward either under-triage or over-triage and range from one to four triage levels, with potentially different clinical implications that were not captured in the present analyses. Future studies should consider methods that preserve both the direction and magnitude of these errors.

Lastly, this was a single-center study, which may limit generalizability to other educational and clinical settings. Multicenter studies are warranted; however, maintaining comparable baseline exposure and minimizing contamination across institutions may be challenging because participating centers may differ in their curricula, prior triage training, and routinely used triage systems.

## Conclusion

Among Thai medical students in a standardized crossover simulation, no statistically significant differences in correct- or under-triage classifications were detected across TRIAGE, ESI, and MOPH; however, the study was not powered to establish equivalence, MOPH showed higher over-triage. TRIAGE consistently required longer completion time than ESI and MOPH, including in restricted-period analyses, supporting an algorithm-specific difference in assessment time. These findings reflect participants’ fidelity in applying each system against its own reference standard and should not be interpreted as evidence of equivalent clinical validity or superiority of any system in real-world triage. Further studies involving trained healthcare professionals and multicenter settings are needed to evaluate performance after structured instruction and in routine clinical practice.

## Supporting information

S1 Table Distribution of predefined case difficulty across the three test sets used in the crossover study. (DOCX)

S2 Table Inter-rater agreement among expert adjudicators for each triage system. (DOCX)

S3 Table Confusion matrices comparing assigned and reference-standard triage levels across the three triage systems. Tables S3a–S3c present the confusion matrices for TRIAGE, ESI, and MOPH, respectively.
(DOCX)

S4 Table Sensitivity analyses of period, system-by-period interaction, and carryover effects for triage outcomes and completion time. (DOCX)

S5 Table Post hoc sensitivity analyses restricted by study period for triage outcomes and completion time. (DOCX)

S1 Fig Thai Rapid Initial Assessment and Grading for Emergency (TRIAGE). Conceptual framework of the TRIAGE system, developed with support from the National Institute for Emergency Medicine, Thailand. The system had not been formally implemented at the time of this study. The figure was independently created for this study and does not reproduce any previously published figure.
(TIFF)

S2 Fig Example of a standardized patient card used in the simulation assessment. From the Thailand Triage Training Kit (TTTK); this material has not been previously published and has no bibliographic reference. Published under a CC BY license with permission from the National Institute for Emergency Medicine (NIEM), Thailand, original copyright 2025.
(TIFF)

S1 Data X. (XLSX)

S1 Checklist CONSORT 2025 Checklist. (DOCX)
