# Supplementary material for: Comparison of three emergency department triage systems among Thai medical students: A randomized three-period crossover simulation study
Source: PLoS One. 2026 Oct 26;21(10):e0350690. doi: 10.1371/journal.pone.0350690 (PMC13704851; doi:10.1371/journal.pone.0350690)
Supplement: Supplementary file 1 [file pone.0350690.s001.docx]

S1 Table. Distribution of predefined case difficulty across the three test sets used in the crossover study.

| Difficulty | Test set 1, n (%) n = 50 | Test set 2, n (%)  n = 50 | Test set 3, n (%)  n = 50 |
| --- | --- | --- | --- |
| Easy | 42 (84.0) | 43 (86.0) | 43 (86.0) |
| Intermediate | 6 (12.0) | 5 (10.0) | 5 (10.0) |
| Hard | 2 (4.0) | 2 (4.0) | 2 (4.0) |
| Difficulty categories were predefined by the three emergency nurses with the highest concordance with the reference answers before random allocation of cases into the three test sets. No significant difference in the distribution of difficulty was observed among the three test sets (exact probability test, P = 0.998). | | | |
