# Supplementary material for: Comparison of three emergency department triage systems among Thai medical students: A randomized three-period crossover simulation study
Source: PLoS One. 2026 Oct 26;21(10):e0350690. doi: 10.1371/journal.pone.0350690 (PMC13704851; doi:10.1371/journal.pone.0350690)
Supplement: Supplementary file 2 [file pone.0350690.s002.docx]

S2 Table. Inter-rater agreement among expert adjudicators for each triage system.

| Triage system | Conger’s weighted kappa | 95% CI |
| --- | --- | --- |
| TRIAGE | 0.900 | 0.892–0.907 |
| ESI | 0.881 | 0.872–0.889 |
| MOPH | 0.871 | 0.862–0.880 |
| Conger's weighted kappa with linear weights was calculated among three independent expert adjudicators for each triage system. CI: confidence interval; TRIAGE: Thai Rapid Initial Assessment and Grading for Emergency; ESI: Emergency Severity Index; MOPH: Ministry of Public Health Emergency Department Triage. | | |
