# Supplementary material for: Comparison of three emergency department triage systems among Thai medical students: A randomized three-period crossover simulation study
Source: PLoS One. 2026 Oct 26;21(10):e0350690. doi: 10.1371/journal.pone.0350690 (PMC13704851; doi:10.1371/journal.pone.0350690)
Supplement: Supplementary file 3 [file pone.0350690.s003.docx]

S3 Table. Confusion matrices comparing assigned and reference-standard triage levels across the three triage systems.

S3a Table. TRIAGE confusion matrix. Distribution of assigned TRIAGE levels according to the reference-standard TRIAGE levels.

| Assigned level | Reference  TRIAGE level 1 | Reference  TRIAGE level 2 | Reference  TRIAGE level 3 | Reference  TRIAGE level 4 | Reference  TRIAGE level 5 | Total |  |
| --- | --- | --- | --- | --- | --- | --- | --- |
| Assigned TRIAGE level 1 | 470  (15.6) | 68  (2.3) | 2  (0.1) | 0  (0) | 6  (0.2) | 546  (18.2) |  |
| Assigned TRIAGE level 2 | 159  (5.3) | 667  (22.2) | 176  (5.9) | 4  (0.1) | 11  (0.4) | 1,017  (33.9) |  |
| Assigned TRIAGE level 3 | 44  (1.5) | 181  (6.0) | 540  (18.0) | 45  (1.5) | 27  (0.9) | 837  (27.9) |  |
| Assigned TRIAGE level 4 | 5  (0.2) | 27  (0.9) | 252  (8.4) | 81  (2.7) | 84  (2.8) | 449  (15.0) |  |
| Assigned TRIAGE level 5 | 0  (0) | 4  (0.1) | 36  (1.2) | 21  (0.7) | 90  (3.0) | 151  (5.0) |  |
| Total | 678  (22.6) | 947  (31.5) | 1,006  (33.6) | 151  (5.0) | 218  (7.3) | 3,000  (100) |  |
| TRIAGE: Thai Rapid Initial Assessment and Grading for Emergency. Values are presented as n (%), with percentages calculated using the total number of card-level assessments for TRIAGE (n = 3,000). Percentages were adjusted for rounding to ensure that totals equaled 100%. | | | | | | | |

S3b Table. ESI confusion matrix. Distribution of assigned ESI levels according to the reference-standard ESI levels.

| Assigned level | Reference  ESI level 1 | Reference  ESI level 2 | Reference  ESI level 3 | Reference  ESI level 4 | Reference  ESI level 5 | Total |
| --- | --- | --- | --- | --- | --- | --- |
| Assigned ESI level 1 | 528  (17.6) | 73  (2.4) | 6  (0.2) | 1  (0.1) | 2  (0.1) | 610  (20.4) |
| Assigned ESI level 2 | 159  (5.3) | 725  (24.2) | 145  (4.8) | 6  (0.2) | 6  (0.2) | 1,041  (34.7) |
| Assigned ESI level 3 | 21  (0.7) | 219  (7.3) | 475  (15.8) | 34  (1.1) | 29  (1.0) | 778  (25.9) |
| Assigned ESI level 4 | 10  (0.3) | 1  (0.1) | 258  (8.6) | 88  (2.9) | 82  (2.7) | 439  (14.6) |
| Assigned ESI level 5 | 2  (0.1) | 2  (0.1) | 16  (0.5) | 51  (1.7) | 61  (2.0) | 132  (4.4) |
| Total | 720  (24.0) | 1,020  (34.1) | 900  (29.9) | 180  (6.0) | 180  (6.0) | 3,000  (100) |
| ESI: Emergency Severity Index. Values are presented as n (%), with percentages calculated using the total number of card-level assessments for ESI (n = 3,000). Percentages were adjusted for rounding to ensure that totals equaled 100%. | | | | | | |

S3c Table. MOPH confusion matrix. Distribution of assigned MOPH levels according to the reference-standard MOPH levels.

| Assigned level | Reference  MOPH level 1 | Reference  MOPH level 2 | Reference  MOPH level 3 | Reference  MOPH level 4 | Reference  MOPH level 5 | Total |
| --- | --- | --- | --- | --- | --- | --- |
| Assigned MOPH level 1 | 469  (15.6) | 108  (3.6) | 3  (0.1) | 2  (0.1) | 2  (0.1) | 584  (19.5) |
| Assigned MOPH level 2 | 124  (4.1) | 682  (22.7) | 174  (5.8) | 23  (0.8) | 4  (0.1) | 1,007  (33.5) |
| Assigned MOPH level 3 | 20  (0.7) | 225  (7.5) | 413  (13.8) | 103  (3.4) | 12  (0.4) | 773  (25.8) |
| Assigned MOPH level 4 | 2  (0.1) | 43  (1.5) | 187  (6.2) | 160  (5.3) | 61  (2.0) | 453  (15.1) |
| Assigned MOPH level 5 | 3  (0.1) | 19  (0.6) | 30  (1.0) | 51  (1.7) | 80  (2.7) | 183  (6.1) |
| Total | 618  (20.6) | 1,077  (35.9) | 807  (26.9) | 339  (11.3) | 159  (5.3) | 3,000  (100) |
| MOPH: Ministry of Public Health Emergency Department Triage. Values are presented as n (%), with percentages calculated using the total number of card-level assessments for MOPH (n = 3,000). Percentages were adjusted for rounding to ensure that totals equaled 100%. | | | | | | |
