# Supplementary material for: Comparison of three emergency department triage systems among Thai medical students: A randomized three-period crossover simulation study
Source: PLoS One. 2026 Oct 26;21(10):e0350690. doi: 10.1371/journal.pone.0350690 (PMC13704851; doi:10.1371/journal.pone.0350690)
Supplement: Supplementary file 4 [file pone.0350690.s004.docx]

S4 Table. Sensitivity analyses of period, system-by-period interaction, and carryover effects for triage outcomes and completion time.

| Outcome | Analysis | P value^†^ | Interpretation |
| --- | --- | --- | --- |
| Correct triage | Period effect | 0.017 | Evidence of a period effect, suggesting that correct triage performance varied across study periods. |
|  | System × period | 0.033 | Evidence of a system-by-period interaction, indicating that differences among triage systems were not constant across study periods. |
|  | Carryover (previous system) | 0.755 | No evidence of a carryover effect. |
| Under-triage | Period effect | 0.062 | No evidence of a period effect. |
|  | System × period | 0.411 | No evidence of a system-by-period interaction. |
|  | Carryover (previous system) | 0.780 | No evidence of a carryover effect. |
| Over-triage | Period effect | 0.458 | No evidence of a period effect. |
|  | System × period | < 0.001 | Evidence of a system-by-period interaction, indicating that over-triage differences among triage systems varied across study periods. |
|  | Carryover (previous system) | 0.080 | No evidence of a carryover effect. |
| Completion time | Period effect | < 0.001 | Evidence of a period effect, suggesting that completion time changed across study periods. |
|  | System × period | < 0.001 | Evidence of a system-by-period interaction, indicating that completion-time differences among triage systems were not constant across study periods. |
|  | Carryover (previous system) | 0.654 | No evidence of a carryover effect. |
| ^†^P values were obtained from adjusted multilevel logistic models for correct triage, under-triage, and over-triage and from an adjusted linear mixed-effects model for completion time. The system-by-period interaction assessed whether differences among triage systems varied across study periods. The carryover analysis included periods 2 and 3 and incorporated the triage system used in the preceding period. | | | |
