# Supplementary material for: Comparison of three emergency department triage systems among Thai medical students: A randomized three-period crossover simulation study
Source: PLoS One. 2026 Oct 26;21(10):e0350690. doi: 10.1371/journal.pone.0350690 (PMC13704851; doi:10.1371/journal.pone.0350690)
Supplement: Supplementary file 5 [file pone.0350690.s005.docx]

S5 Table. Post hoc sensitivity analyses restricted by study period for triage outcomes and completion time.

| Outcome | Primary analysis: overall system effect,  P value | First-period-only analysis: system effect, P value | Excluding period 3: system effect,  P value | Interpretation |
| --- | --- | --- | --- | --- |
| Correct triage | 0.124 | 0.328 | 0.859 | No overall system effect in the primary, first-period-only, or period 3-excluded analyses; however, evidence of a system-by-period interaction was observed. |
| Under-triage | 0.449 | 0.663 | 0.630 | No overall system effect and no evidence of system-by-period interaction across sensitivity analyses. |
| Over-triage | < 0.001 | 0.270 | 0.623 | Overall system effect was observed in the primary analysis, but not in first-period-only or period 3-excluded analyses; evidence of system-by-period interaction was observed. |
| Completion time | < 0.001 | 0.001 | < 0.001 | Overall system effect remained consistent across all sensitivity analyses, with evidence of system-by-period interaction. |
| Primary analysis included all three study periods and evaluated the overall effect of triage system while adjusting for period, sequence, and academic year. The first-period-only analysis was restricted to period 1, whereas the analysis excluding period 3 included periods 1 and 2. Classification outcomes were evaluated using multilevel mixed-effects logistic regression models with crossed random intercepts for participant and patient card; completion time was evaluated using a linear mixed-effects model with a participant-level random intercept. All restricted-period assessments were post hoc exploratory analyses conducted to evaluate the robustness of the primary findings to temporal patterns. | | | | |
